# Age-Related Changes in the Mechanical Properties of Human Fibroblasts and Its Prospective Reversal After Anti-Wrinkle Tripeptide Treatment

**DOI:** 10.1007/s10989-013-9370-z

**Published:** 2013-09-18

**Authors:** Ida Dulińska-Molak, Monika Pasikowska, Katarzyna Pogoda, Małgorzata Lewandowska, Irena Eris, Małgorzata Lekka

**Affiliations:** 1 Faculty of Materials Science and Engineering, Warsaw University of Technology, Wołoska 141, 02507 Warsaw, Poland; 2 Dr Irena Eris Cosmetic Laboratories, Centre for Science and Research, Puławska 107a, 02595 Warsaw, Poland; 3 The Henryk Niewodniczański Institute of Nuclear Physics, Polish Academy of Sciences, Radzikowskiego 152, 31358 Krakow, Poland

**Keywords:** Atomic force microscopy, Depth-sensing analysis, Tripeptide, Fibroblasts, Cell cytoskeleton

## Abstract

One of an essential characteristic of human skin are time dependent mechanical properties. Here, we demonstrate that stiffness of human dermal fibroblast correlates with age and it can be restored after anti-wrinkle tripeptide treatment. The stiffness of human fibroblasts isolated from donors of 30-, 40- and 60 years old were examined. Additionally the effect of anti- wrinkle tripeptide of latter cells was investigated. The atomic force microscopy measurements were performed on untreated fibroblast as well as on treated with the peptide. The Young’s modulus for two indentation depths 200 and 600 nm of each cell type was determined. The Young’s modulus increases with age of the cells. The highest values of Young’s modulus were obtained for fibroblasts collected from 60 years old donors, for indentation depth of ~200 nm. For larger indentation depth of 600 nm there are no significant differences in stiffness between cells. Fibroblasts treated with the anti-wrinkle tripeptide exhibit lower Young’s modulus. The cells derived from 40- and 60-years old donors restored stiffness characteristic to the level of 30 years old subjects. The results show correlation between stiffness and age of the human fibroblast as well as impact of anti-wrinkle tripeptide on the mechanical properties of skin cells.

## Introduction

Aging refers to a complex of physical, biological, and biochemical processes that lead to dysfunctions of molecules, cellular structures, and organs. One of the essential characteristic of the aging process of human skin is changes in its mechanical properties. Previous studies have shown that biomechanical properties are important for cell functions (Janmey and Weitz 2004). Despite this, there is little information about the relationship between aging and the mechanical properties of cells.

The determination of mechanical properties of living cells has become possible with the development of local measurement techniques such as atomic force microscopy (AFM). Since its discovery, distinct cell types have been investigated encompassing blood or cancer cells measured in various aspects of human pathologies (Lim 2006; Dulińska et al. 2006; Suresh 2007). Among many directions of biomechanical studies, skin fibroblasts and keratinocytes have been analysed (Berdyyeva et al. 2005; Kobiela et al. 2013). Results obtained by Berdyyev’a et al. showed that the human foreskin epithelial cells became significantly more rigid during ageing. Moreover, Kobiela et al. presented interesting results on stiffness decrease in living epidermal keratinocytes treated with sodium lauryl sulphate.

Fibroblasts are major cell type present in the dermis, which are capable of producing collagen fibers—the main component of the extracellular matrix ECM of the dermal connective tissue. Simultaneously it is believed that fibroblasts connected to collagen network provide dermal support for the epidermis and give rise to elastic properties of a tissue (Sorrell and Caplan 2004; Metcalfe and Ferguson 2007). Mechanical properties of the skin change not only with aging but also as the reason of exposure to various environmental factors like UV irradiation and air pollutions. This leads to decrease in synthesis of collagen as well as damages due to processes like glycation or oxidative stress (Woods et al. 2012; Poljsak et al. 2012). For that reason, the effect of anti-wrinkle peptides, on cells elastic properties is essential to be understood. In particular, a molecule known as stimulator of collagen synthesis in human fibroblasts is important since the stimulation occurs via activation of TGF-β (Transforming Growth Factor), a key element in the synthesis of collagen fibers (Pentapharm 2013; Kim et al. 2007). Moreover, it has been demonstrated that mechanical properties of cells and the state of the cytoskeleton organization affect the interaction between fibroblasts and the ECM (Walpita and Hay 2002), so mechanical changes may reveal new aspects of the age-related degradation of the elastic properties of the dermis. For this reason skins’ elastic recovery after anti-wrinkle tripeptide treatment seems to be important to be examined.

Here, we show that individual dermal fibroblasts exhibit a change in elasticity during aging in vivo. The experiment was performed on dermal fibroblasts isolated from skin slices taken from human donors aged 30–60. In our studies, using the AFM method, the relative Young’s modulus (a parameter describing the elastic properties of cells) was calculated for two indentation depth values, smaller ~200 nm and bigger ~600 nm. The choice was dictated by results described by Pogoda et al. (2012) showing that for small indentation depths, the Young’s modulus describes the regions rich in the network of actin filaments while for larger indentation depth, the overall stiffness of a whole cell is obtained.

Our results of depth-sensing analysis of the mechanical properties of living fibroblasts, was performed under physiological conditions with and without presence of anti-wrinkle tripeptide. This enables us to describe the non-homogeneity of the cell cytoskeleton, particularly, its contribution linked to actin filaments located beneath the cell membrane. We observed the large differences between modulus value of cells from three types of patients for small indentation whereas large indentations reveal very similar character of modulus distribution.

## Materials and Methods

### Cell Lines

The studies were carried out on primary human dermal fibroblasts (generated from separated dermis from 30-, 40- and 60-years old volunteers) cultured in Eagle’s medium (MEM; Sigma-Aldrich) supplemented with 10 % fetal calf serum, 10 mM HEPES, 2 mM l-glutamine and antibiotics (100 U/ml penicillin; 0.25 g/ml streptomycin sulfate). Passages between 2 and 8 were used. All cells were cultured in culture flasks then trypsinized using trypsin–EDTA solution (0.05 %; Gibco), diluted ten times in growth medium and spread on glass coverslips (Menzel Gläser, 18 × 18). Prepared cells were treated with 0.1 % of solution of anti-wrinkle peptide (palmitoyl-lysyl-valyl-lysine bistrifluoracetate salt; solution containing 900–1,100 ppm of peptide; Pentapharm 2013) for 5 days. Concentration of the solution was chosen based on proliferation assays (data not shown). Cells were kept at 37 °C in an atmosphere of 95 % air/5 % CO_2_ and afterwards were taken for AFM measurements.

### Fluorescence Microscopy

The organization of actin cytoskeleton was visualized using fluorescent dyes in a following way. First, cells grown on a coverslips, were fixed with 3.7 % paraformaldehyde (Sigma, dissolved in the PBS buffer) for 20 min, then rinsed two times in a pure PBS buffer and permeabilizated with 0.2 % Triton X-100 solution in 4 °C for 4 min (Sigma, in the PBS buffer). Then, they were again rinsed in the PBS buffer. Actin filaments were stained using the solution containing phalloidin labeled with Alexa Fluor 488 (1:200, PBS solution, Molecular Probes, absorption *λ* = 495 nm, emission *λ* = 518 nm) for 30 min incubation at room temperature. Next, the excess of dye was removed by rinsing coverslips in the PBS buffer. Cells nuclei were stained using Hoechst 34580 solution (1:500, PBS solution, Molecular Probes, absorption *λ* = 380 nm, emission *λ* = 460 nm) for 15 min incubation also in room temperature. At the end cells were rinsed in the PBS buffer for 5 min. All fluorescence measurements were taken directly after staining and performed using Olympus IX71 microscope equipped with a 100 W mercury lamp. Images were recorded using the CCD camera (XC10 digital camera) mounted on an optical microscope Olympus IX71.

### Atomic Force Microscopy

Measurements of cell elastic properties were performed using a commercial microscope (model XE-120; Park Systems, Korea. The silicon nitride cantilevers MLCT (Bruker) were used as probes. This type of cantilever was characterized by a spring constant of 0.01 N/m. The shape of the AFM tip is a four-sided pyramid with height 2.5 μm and radius of curvature about 20 nm. Fibroblasts were measured in MEM buffer. All measurements were performed at room temperature.

#### Data Acquisition

Force curves were collected from randomly chosen cells. Imaging was performed before elasticity measurements. Cell topography was recorded at a scan rate between 1.0 and 1.5 Hz and a set point in the range 0.3–0.5 nN. Scan size of 90 × 90 μm (256 × 256 pixels) was chosen to visualize whole fibroblasts. On average, 100 curves for each single cell were recorded (about 20 cells, were measured for each age).

#### Cell Stiffness Determination

**Fig. 1:**
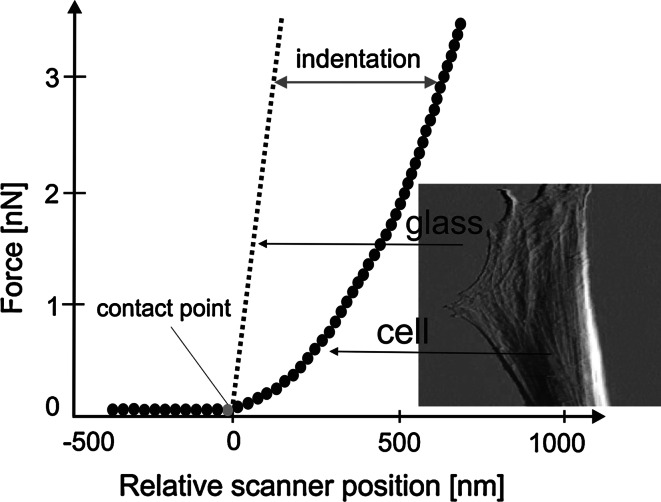
The idea of the Young’s modulus determination based on the subtraction of curves recorded on stiff (in our case glass coverslip) and compliant (dermal fibroblasts) surfaces

Cell stiffness is determined on the basis of the *force versus indentation* curve that is usually obtained by subtraction of cantilever deflections measured on stiff and compliant surfaces at a given, relative sample position (Fig. 1). When a stiff material (not easily deformable, for example glass) is investigated, the deflection reflects the position of the sample. This is represented by a straight sloped line which is usually used as a reference line (needed for calibration). For compliant samples, for example cells, cantilever deflections are much smaller and the resulting force curve has non-linear character. The difference between these curves determines the deformation of the sample surface. Depending on the magnitude of the indentation depth, distinct properties can be studied, revealing the heterogeneity of the structure of the cell interior.

The force versus indentation curve describes the mechanical response to the applied load, which is characteristic for each material. The relative Young’s modulus value, characterizing the cell stiffness, can be evaluated in the framework of Hertz contact mechanics (Domke and Radmacher 1998), taking into account an infinitely stiff indenter with a selected geometry of the AFM tip (i.e. spherical, parabolic, conical, or flat-ended) and a flat, deformable substrate (Sneddon 1965). Usually, the AFM probe tip is a four-sided pyramid that can be modeled either by a cone or by paraboloid. Thus, two formulas are usually used to describe the relationship between the load force and the resulting indentation depth:

- for a conical tip

1 $$ F(\delta ) = \frac{2 \cdot \tan \alpha }{\pi } \cdot E_{eff} \cdot \delta^{2} $$

- for a parabolic tip

2 $$ F(\delta ) = \frac{4}{3} \cdot \sqrt R \cdot E_{eff} \cdot \delta^{1.5} $$

where *F* is the loading force, *α* the open angle = 35°, *R* the radius of curvature of the AFM tip = 20 nm, *E*_*eff*_ the reduced Young’s modulus, and *δ* the indentation depth. The reduced Young’s modulus is given by:

3 $$ \frac{1}{{E_{eff} }} = \left( {\frac{{1 - \mu_{tip}^{2} }}{{E_{tip} }} + \frac{{1 - \mu_{samp}^{2} }}{{E_{samp} }}} \right) $$

when *Es*_*ample*_ < < *E*_*tip*_ (as is true for living cells) then:

4 $$ E_{eff} = \frac{{E_{samp} }}{{1 - \mu_{samp}^{2} }} $$

where *μ*_*sample*_ and μ_tip_ are the Poisson ratios, related to the compressibility of the sample material, ranging from 0 to 0.5. The Poisson ratio for cells is difficult to determine, therefore, all calculations must assume its value. Very often this value is set to be equal 0.5 because cells can be treated as the incompressible material. The final Young’s modulus of a cell was calculated taking into account all values determined for the whole set of force versus indentation curves recorded for a single cell.

The force–indentation curves, obtained for all studied cell types, were analyzed assuming that the shape of the AFM probe is a cone. The choice of the model was dictated by χ^2^values describing the goodness of the fit. They were smaller for the model with a conically shaped AFM tip than when a parabolic shape was assumed. After choosing the contact point position, the fit was performed at different indentation depths.

### Statistical Significance

Data were expressed as *mean* *±* *standard deviation* (*SD*). Statistical significance was identified by Student’s *t* test or one-way analysis of variance for the difference among pairs. A *P* value <0.05 was considered to be statistically significant.

## Results

### Cell Proliferation and Spread Area

**Fig. 2:**
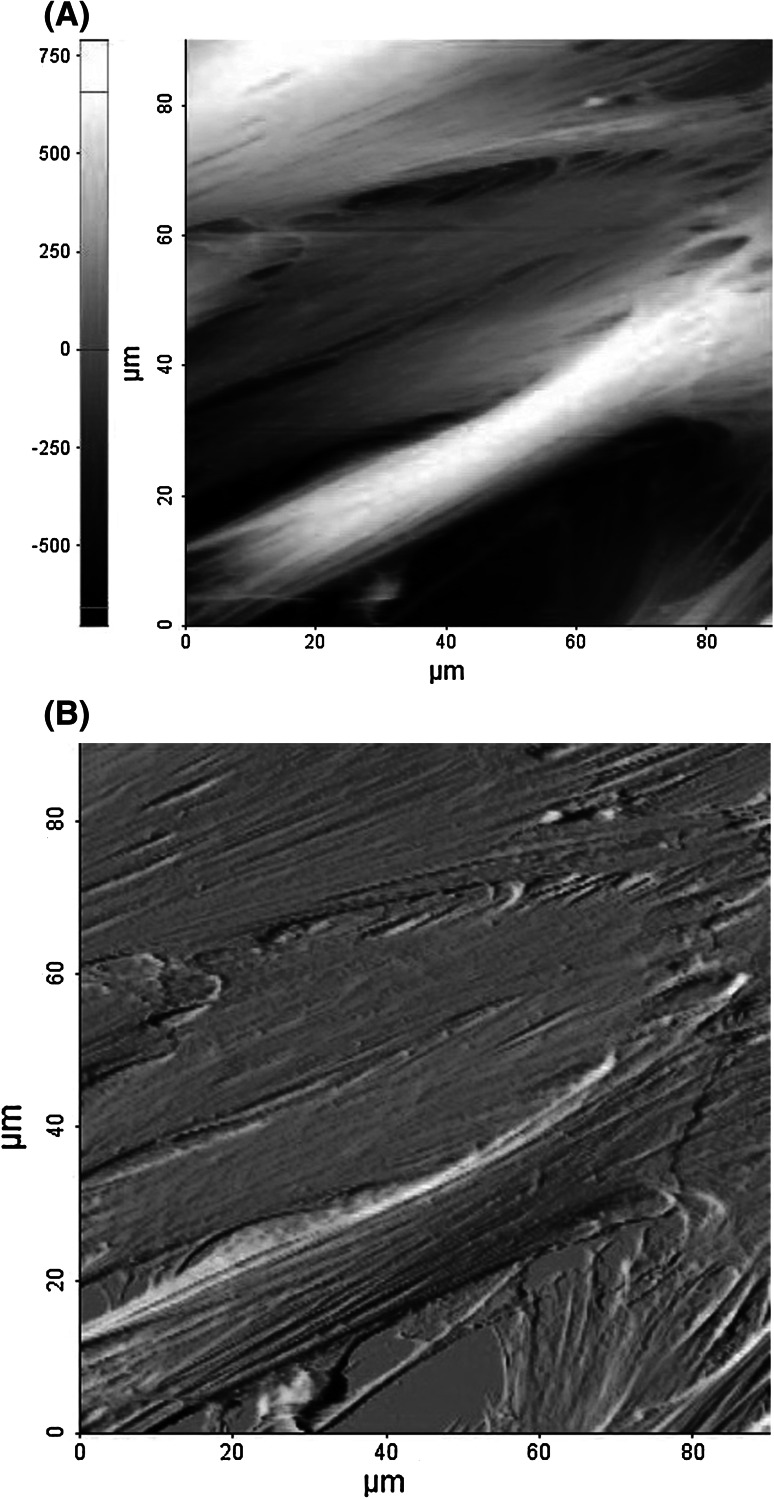
Typical surface topography of fibroblasts. **a** contact height **b** and deflection images were recorded before treatment with anti-wrinkle peptide

**Fig. 3:**
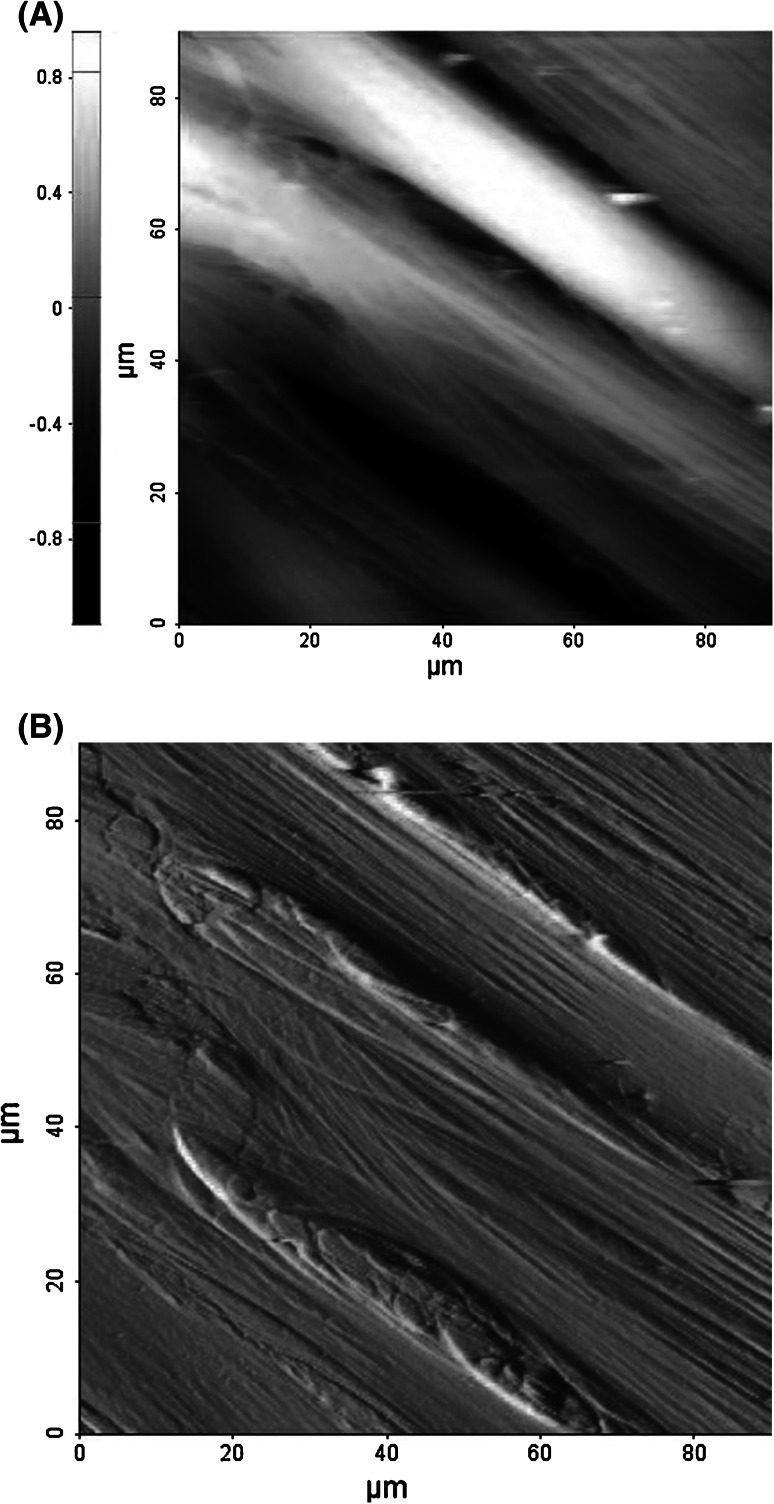
Typical surface topography of fibroblasts. **a** contact height **b** and deflection images were recorded after treatment with anti-wrinkle peptide

**Fig. 4:**
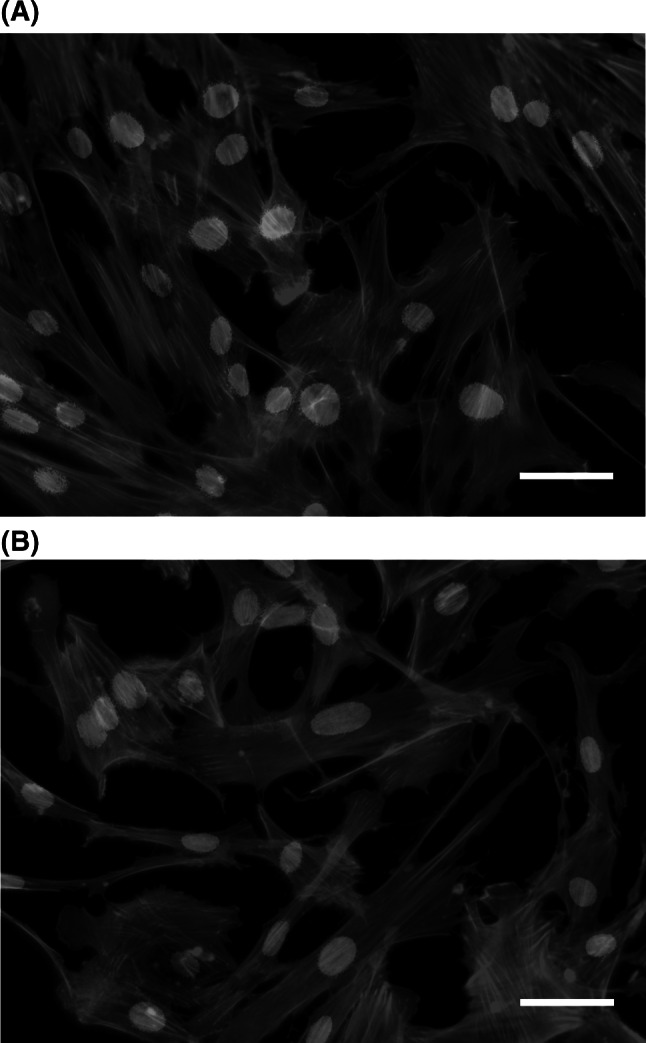
Actin filament (*green*) distributions. **a** non-treated and **b** anti-wrinkle peptide treated skin fibroblasts generated form 40 years old volunteer. *Blue* denotes cell nuclei stained with Hoechst; *scale bar* = 100 μm (Color figure online)

The morphology of two studied samples, i.e. living fibroblasts before and after treatment with anti-wrinkle peptide was studied using both atomic force and fluorescent microscopes (Figs. 2, 3, 4).

Cells, which were grown for AFM elasticity measurements show no significant difference between treated and non-treated fibroblasts. Both samples showed elongated shape of closely packed cells with the presence of long stress fibers on their surface (Figs. 2, 3). Number of cells as well as their area of spreading remains constant.

Since the AFM can image only the cell surface, the fluorescence staining of actin filaments has been performed in order to evaluate their organization (Fig. 4). Similarly to AFM images, fibroblasts displayed comparable morphological features indicating that treatment with anti-wrinkle peptide does not change cells appearance leading to their impaired functioning.

To quantify the cell morphology, cell spread area has been calculated in a following way. First, fluorescent images were binarized. Then, both the area occupied by all cells and the number of cell nuclei has been determined (both quantities were calculated using standard procedures with image processing and analysis software package “ImageJ” (http://rsbweb.nih.gov/ij). The ratio between these quantities enabled to estimate the average area taken by the single cell, which is 12,150 ± 2,420 m^2^ and 11,430 ± 2,450 μm^2^ for reference (untreated) and treated fibroblasts, respectively. Also cells amount remain constant in both cases. After treatment with anti-wrinkle peptide, the cell area decreased of about 6 % indicating slight effect of the compound on cell spread area leading to cell shrinking. This leads to a conclusion that tested anti-wrinkle peptide does not stimulate cell proliferation (what is closely linked with accelerated cell death) but only collagen production.

### Fibroblasts Elastic Properties

**Fig. 5:**
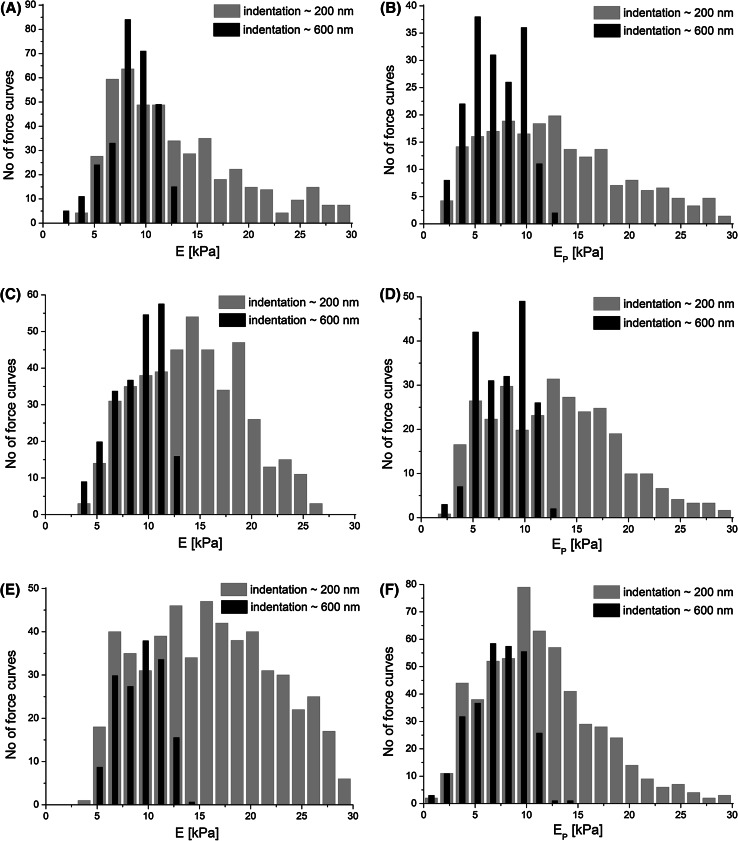
*Histograms* of the relative values of the Young’s modulus obtained for fibroblasts. (**a** and **b** age = 30, **c** and **d** age = 40, **e** and **f** age = 60) before (**a**, **c**, **e**) and after treatment with anti-wrinkle peptide (**b**, **d**, **f**). Moduli distributions obtained for indentation depths: 200 nm (*gray columns*) and 600 nm (*black columns*)

In our studies, the relative Young’s modulus was calculated for two constant indentation depths of 200 and 600 nm, representing the border limits of the measured values of the indentation depths (Fig. 5). Depending on the indentation range, distinct characters of the Young’s modulus distribution were observed. The widest distribution was obtained for small indentation (*δ* = 200 nm, grey columns in Fig. 4) where the width of the distribution was above 15 kPa (*n* = 500 force curves), taken as the full width taken at half height (FWHH). For larger indentation (δ = 600 nm, black columns in Fig. 4) furnished histograms almost half as wide as that for small indentation (FWHH = 7.5 kPa, *n* = 250 force curves). The narrower distribution was observed for all cells with FWHH within the range of 1–6 kPa, independently of the fibroblasts treatment with anti-wrinkle peptide.

Thus, at large indentations, the distribution of the Young’s modulus for all cell populations regardless of age were again narrower than those obtained for small indentations. Furthermore, the increase of the indentation depth lowered the mean of the modulus irrespective of the measured sample type (Table 1).

**Table 1:** Young’s modulus calculated for fibroblasts originated from the skin donated by three volunteers at age 30, 40, 60 years before and after treatment peptides and treated with anti-wrinkle tripeptide (denoted by *letter* *P*)

Sample	E_200_ (kPa) indentation depth = 200 nm			E_600_ (kPa) indentation depth = 600 nm		
	Mean	St. dev.	SE mean	Mean	St. dev.	SE mean
Age-30	12.68	4.21	0.32	8.62	1.84	0.17
Age-30 (P)	12.23	5.23	0.41	7.06	1.62	0.22
Age-40	13.84	3.72	0.25	9.09	1.88	0.21
Age-40 (P)	12.33	5.16	0.31	7.91	1.65	0.24
Age-60	16.59	4.81	0.32	9.33	1.61	0.16
Age-60 (P)	11.43	5.02	0.33	7.36	1.72	0.17

*SEM* is a standard error of the mean

**Fig. 6:**
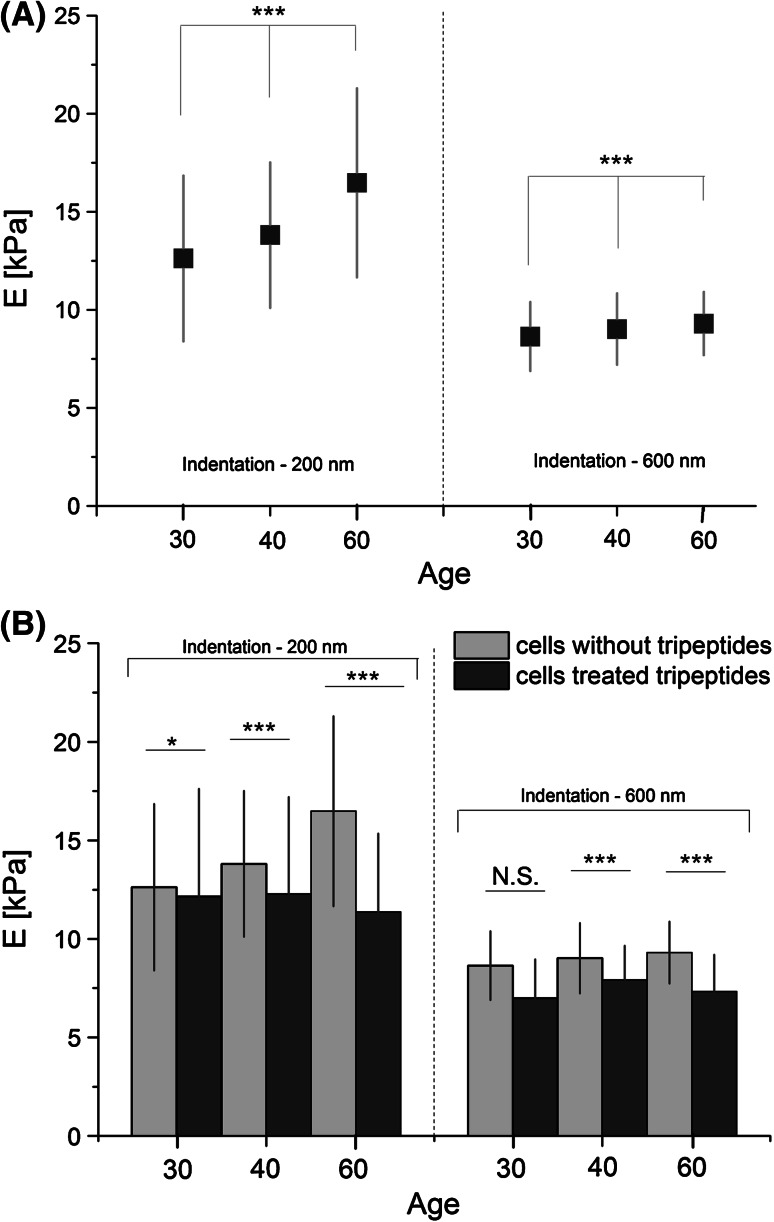
**a** The Young’s modulus (E) determined for fibroblasts at the indentations depths of 200 and 600 nm. Data are presented as mean ± standard deviation. *** *P* ≤ 0.001. **b** Mean values of Young’s modulus (E) determined for two indentation depths of 200 and 600 nm for fibroblasts collected before and after treatment with anti-wrinkle peptide expressed as a mean ± standard deviation (*** *P* ≤ 0.001, * *P* ≤ 0.05, *N.S.* no significant difference)

In our analysis, the data were calculated assuming the conical shape of the probing AFM tip. Figure 6a shows a relationship between the Young’s modulus compared for two indentations depths i.e. 200 and 600 nm, calculated for fibroblasts taken at a given age (30, 40, 60 years).

The results reveal that there are significant differences between the fibroblasts stiffness (i.e. Young’s modulus) collected from patients at different age. In case of small indentation depth equaled to 200 nm, the largest value of Young’s modulus was obtained for fibroblasts collected from 60 years old volunteer. Moreover, the Young’s modulus decreases as the age of volunteers drops. For indentation depth of 600 nm, the effect is not pronounced, nevertheless, still the tendency of smaller Young’s modulus values for younger persons is observed. The statistical significance was verified using the one-way analysis of variance, which shows significant difference among all studied sample types (*P* ≤ 0.001).

The obtained results show that the observed difference between Young’s moduli for smaller indentation depths allows the detection of age-related changes, while the mechanical response from deeper parts of a cell indicates mechanical similarity of fibroblasts between studied samples.

### Influence of Anti-Wrinkle Peptide

The comparison of fibroblasts elastic properties performed for samples as a function of age, cultured in medium with and without anti-wrinkle peptide, is presented in Fig. 6b.

Results show that presence of tripeptide influenced the Young’s modulus irrespective of the age of the studied samples and indentation depth. In general, one can see that elastic moduli of fibroblasts cultured in presence of peptide decreases. The data analysis performed for from small and large indentation depths show that the most significant change in modulus is observed for fibroblasts originating from older skin (60 years) as compared to those from the youngest studied sample. This indicates the strongest impact of the peptide to fibroblast mechanical properties derived from skin of the 60-year old volunteer. For larger indentations (600 nm), the modulus variations between samples are less prominent but still analogous tendency as for small indentations are observed. There is no statistically significant difference of cell’s elasticity for the sample collected from 30 year old women. With the age increasing, slightly larges effect of the tripeptide is detected leading to better statistical significance (*P* ≤ 0.001).

## Discussion

The outer part of a cell comprises a cell membrane, then actin cortex with the anchored endings of microtubules and intermediate filaments (Rotsch and Radmacher 2000; Janmey 1991). The structure of this layer contains a set of accessory proteins that can regulate the properties, functioning and structure of cytoskeleton fibres. These proteins can polymerize, cross-link, and bundle all filamentous structures forming a cell cytoskelelton. Rotsch and Radmacher (2000) demonstrated that the actin cytoskeleton plays a major role in a cell’s mechanical stability, measured by AFM, by adding cytoskeleton-disrupting drugs and measuring the impact on cell elasticity. Berdyyev’a et al. showed a correlation between the density of the cytoskeleton and cell rigidity in aging epithelial cells (Berdyyeva et al. 2005). Early work on the cytoskeleton of human peripheral blood lymphocytes showed that the concentration of F-actin fibres increases in older cells (Radmacher 1997). Recent work on the measurement of elasticity of rat liver macrophages (Tsukruk et al. 2000) and transformed mouse fibroblasts (Vinckier and Semenza 1998) using AFM demonstrated that actin fibres have a considerable influence on cell elasticity. It has been also postulated that the age-associated loss of cell flexibility is rooted in an altered polymerization of the actin cytoskeleton in the optical deformability of the aging cells compared to young fibroblasts (Schulze et al. 2010). Our results point out that elastic properties of skin fibroblasts showed age-related changes in a thin, superficial layer of a cell (the Young’s modulus increases when determined for indentation depth of 200 nm is increasing with cell aging. Larger indentations of 600 nm showed no differences, Table 1; Fig. 4). This is in agreement with the results presented by Schulze et al. showing age-related stiffening of skin fibroblasts. Therefore, by adding that anti-wrinkle tripeptide we hypothesise that elastic properties should increase. A drop of the Young’s modulus was expected since smaller moduli value denote higher deformability of cells (Lekka et al. 2012). Such result was observed after treatment with anti-wrinkle peptide indicating that actin filament polymerization reaches the same level independently of the age of a healthy donors with modulus value between 12 and 11 kPa.

The age-related impairment of flexibility of the skin can origin from the interplay between decreased density and altered organization of collagen fibres forming an elastic network, and reduced amount of proteoglycans (Uitto 2008; Pierard and Lapie’re 1977; Lavker et al. 1987). The consequences of those changes are atrophies and elastosis in dermis, which is exhibited by showing up of wrinkles (Uitto 2008; Yasui et al. 2013). Thus the effect of anti-wrinkle peptide, i.e. palmitoyl-lysyl-valyl-lysine bistrifluoracetate salt, on fibroblast stiffness was investigated. Most probably its effect relies on stimulation of collagen production by activation of TGF-β growth factor, responsible for procollagen synthesis by fibroblasts (Pentapharm 2013). Figure 5b shows the increase of the Young’s modulus for tripeptide-treated fibroblasts as a function of age. The significant stiffness variations were obtained for 200 nm indentation what strongly suggests that anti-wrinkle peptide affects mostly structures distributed beneath cell membrane like actin fibres. Reorganization of actin, due to tripeptide activity, could have a direct effect on organization and mechanical properties of the collagen matrix since the actin cytoskeleton is an essential component of the interaction between fibroblasts and the ECM (Walpita and Hay 2002). Also rheological measurements used to characterize fibroblast-populated collagen gels as dermis equivalents showed the influence of changes in the mechanical properties of cells and the cytoskeleton on the organization and mechanical behaviour of the collagen matrix (Iordan et al. 2010). As a result, selected peptides may be effective in the fight against the signs of aging skin cells.

## Conclusions

Molecular markers based on altered pattern of genes or proteins have been used to monitor aging processes. However, the mechanical properties of cells are linked to their cytoskeletal structure, which in turn is governed by cellular function. The capability of the AFM technique to measure mechanical properties at a local scale enabled to trace differences among fibroblasts isolated from skin of healthy, 30-, 40- or 60-years donors. The presented results clearly shows that there is a correlation between the age of a women and elastic properties of fibroblasts what points out the use of cell’s elasticity as a potential biomarker of skin aging on the cellular level. We suggest that measurement of the mechanical properties of individual cells also offers a novel, to our knowledge, cytological alternative to the genomic and proteomic techniques characterizing cellular senescence as well as influence of active components like drugs or cosmetic ingredients.
